# The Value of Compression of the Aorta in the Treatment of Post-Partum Hemorrhage

**Published:** 1907-06

**Authors:** F. Percy Elliott

**Affiliations:** Medical Officer to the Walthamstow Public Dispensary.


### THE VALUE OF COMPRESSION OF THE AORTA IN THE TREATMENT OF POST-PARTUM HEMORRHAGE.

BY

F. PERCY ELLIOTT, M.B.,

*Medical Officer to the Walthamstow Public Dispensary.*

---

SINCE Mr. Stanmore Bishop[1] and Dr. Le Page[2] recently emphasised the value of compression of the aorta in post-partum hemorrhage, much discussion has taken place as to its practical utility. The following case occurred in private practice, and is of interest if only in regard to the value of compression of the aorta :—

Mrs. L., æt. 22 years. Third pregnancy. Previous pregnancies normal. The last child died at birth, owing to the umbilical cord being round its neck, proper assistance not being available.

The patient was well nourished and free from organic disease, but was of a highly neurotic temperament. There was no history

[1] *Practitioner*, 1906, lxxvii. 145. [2] *Brit. M. J.*, 1907, i. 185.

of hæmophilia. No hemorrhage had occurred during pregnancy.

The present labour began on March 3rd and was preceded by sharp uterine hemorrhage shortly before the "pains" began. The patient was first seen at 9 p.m., and the state of affairs then was as follows: feeble and irregular labour pains, slight bleeding, os dilated sufficiently to admit one finger, lower margin of placenta overlapping os, position of fœtus normal, pulse normal, general condition of patient good.

At 11.30 p.m. on the same day profuse hemorrhage occurred. At this time labour pains were strong and frequent. The os admitted the tips of three fingers. The pulse rate had risen to 120. It was decided to turn and deliver the child. Under chloroform the os was gradually dilated with the fingers and hand, and the child turned and delivered. Delivery took place at 12.45 a.m., the child being alive and full term. Very little bleeding occurred during dilation and version, and ceased as soon as the first leg was brought down through the os. Immediately on delivery of the child sharp bleeding followed, and the uterus then began to show signs of inertia. The pulse rate had risen to 130 when the child was born, and with this further bleeding soon reached 140. Manual compression of the aorta, however, immediately stopped the bleeding, and with returning uterine contractions the placenta was expressed, and without difficulty, half an hour after delivery of the child. The uterine cavity was then explored and emptied of a few blood-clots. There were no placental remains. The cervix was not torn through. After the uterus was emptied, and a drachm of ergot and some nourishment were given, a little friction soon succeeded in getting the uterus firmly contracted. The general condition of the patient had, however, become very serious. Her pulse had risen to between 140 and 150, and was threadlike. She was very pale, and complained of coldness, faintness and nausea. She was quickly packed with hot-water bottles, covered with blankets, and her head lowered. As soon as nausea became less, more nourishment was given and retained. Compression of the aorta was relinquished soon after the uterus had become firmly contracted.

Three hours after delivery there was marked improvement in all the symptoms. The pulse had dropped to 120, was much stronger, and there was no faintness or nausea. The face had regained some colour, the patient was warm and had taken about half a pint of nourishment and retained it all. She complained chiefly of thirst and tiredness. The uterus was still firmly contracted, and no bleeding had occurred since compression of the aorta had been discontinued.

At 10 a.m. her condition again became very serious. Bleeding had returned shortly before this, and the uterus had become large and flabby. Alarming symptoms of shock were now present.

The pulse at the wrist was scarcely perceptible, and too rapid to be counted. The patient complained of being cold and faint, her body being covered with clammy sweat. She soon began to get very restless, and to throw her arms about and cry for air. She then suddenly became cyanosed, had a convulsion and became unconscious. An hypodermic injection of strichnine was immediately given, and in a few minutes consciousness returned, but lasted only momentarily, the patient relapsing into a comatose condition, her pulse now disappearing at the wrist and respiration ceasing. The uterus, which extended above the navel and was full of blood-clots, was in the meantime emptied and compressed through the abdominal walls. There was no response to stimulation, and no time was lost in applying pressure to the abdominal aorta. Respiration had just stopped when the aorta was compressed, and this blood-vessel could be felt pulsating feebly. Pressure was applied with the clenched fist through the abdominal walls and the uterus.

Respiration began again in less than a minute after the aorta was compressed. Subcutaneous transfusion of normal salt solution was then begun. While transfusion and compression of the aorta were being carried out, the patient's legs were elevated and firmly bandaged from the insteps to the groins, and a hypodermic injection of "ernutin" was administered. The head of the patient, already low, was further lowered by elevating the foot of the bed on two chairs. A pint of saline fluid was rapidly transfused beneath the skin of the inframammary region on one side, and a similar quantity in the same region of the other side. The site of injection was then changed to the right flank, as both inframammary regions were bulging with fluid, owing to rapid transfusion and slow absorption. The aorta began to fill out when about a pint of fluid had been absorbed, and before two pints of fluid had been absorbed the patient regained consciousness, and the aorta could be felt pulsating strongly against the fist. Coincidently with the rise in blood-pressure, improvement in all the symptoms took place, the pulse reappearing at the wrist when between two and three pints of fluid had been absorbed. The patient then complained of being hot and thirsty, and soon began to perspire. As soon as consciousness was fully restored, friction and compression were applied to the uterus, and the relaxed organ then responded quickly, although these measures previous to compression of the aorta failed to produce contraction. Pressure was then applied through the abdominal wall directly on the aorta. For five hours life ebbed and flowed in a most remarkable way. During the whole of this time compression of the aorta was kept up continuously by my assistant and myself. After the pulse had reappeared at the wrist transfusion was carried on intermittently, as the tendency to relapse from shock threatened. At 6 p.m. the state of affairs had materially improved. The pulse had got down to 130, and was fairly full. The uterus was firmly contracted, and had shown no tendency to relaxation again. Not a single drop of blood had escaped *per vaginam* since compression of the aorta had been applied, and the uterus had been finally got to contract, and this, notwithstanding the fact that the uterus had been repeatedly squeezed to ascertain whether any blood was escaping *in utero*. The patient complained now chiefly of pain in her legs due to constriction of the bandages, and also of great thirst and weariness. Since she had become conscious about 6 oz. of nourishment had been taken, and this had all been retained. Transfusion was entirely stopped at 6 p.m. During five and a half hours between ten and twelve pints of fluid were transfused. The needle was not removed from the flank until transfusion was finished, the receiver full of fluid being kept at the same level as the flank when transfusion was temporarily suspended. After removing the needle a piece of adhesive plaster was placed over the wound, about two pints of fluid being still unabsorbed when the needle was removed.

The legs of the patient were kept suspended for about half an hour altogether.

Between 11 and 12 p.m. the condition of the patient was eminently satisfactory. The pulse had got down to 100, and was strong and fairly full. The uterus was firmly contracted, and the diaper, which had been applied immediately after compression of the aorta and emptying of the uterus at 11.30 a.m., was unsoiled. All saline fluid had been absorbed. The patient had slept, taken and retained all nourishment, and had regained some colour. She complained, however, of great pain in her legs and of insatiable thirst. Otherwise she said she felt altogether better. The bandages were then very carefully removed, and save for pain and throbbing in her legs, no ill effects followed removal. The legs had been kept warm by hot-water bottles ; the foot of the bed was still kept elevated. The patient was left about an hour after removal of the bandages, and from every point of view was in a very satisfactory condition.

What promised to be a remarkable recovery from post-partum hemorrhage and shock terminated unfortunately in a most unexpected and tragic manner. At 2 a.m., the patient craving for drink, and finding she was refused more than the prescribed quantity, watched her opportunity, and the nurses leaving her bedside for a moment, she sprang out of bed for some water on a table near, and fell in a collapsed state on the floor. In spite of everything, she soon succumbed to heart failure. At the time of death the uterus was still firmly contracted, and less than a tablespoonful of blood had escaped *per vaginam* since she was seen at midnight.

*Remarks.*—Although this patient died, the great value of compression of the aorta was well illustrated. The immediate cause of death was undoubtedly heart failure consequent on the exertion in getting suddenly out of bed, and sudden heart failure is by no means uncommon, even after far less hemorrhage and shock than occurred in this case. Bleeding had ceased for nearly fourteen hours before the patient died, and at the time of death no further bleeding took place. Shock also was being rapidly recovered from.

It is quite obvious in the circumstances attending this case, at the time when compression of the aorta was adopted the second time, that none of the methods of treating atonic post-partum hemorrhage advocated in text-books could have been relied on here for rescuing the patient from immediate death. The patient must have died before plugging of the uterus or the application of perchloride of iron could have been performed, even if these means would eventually have stopped bleeding, a result which admittedly is not always obtained. Compression of the uterus itself in the manner advocated by Dr. Herman[1] would, if carried out continuously for some hours, have prevented further bleeding. But compression of the uterus by this method (which is undoubtedly the best) cannot be carried out continuously for any great length of time, a fact which Dr. Herman himself admits.[2] Further loss of even a small quantity of blood, as must have occurred in changing compression with an assistant and in again changing when the latter became tired, was a matter of vital importance during at least the four first hours that the aorta was compressed. During this period the patient repeatedly relapsed in spite of no bleeding taking place, and the uterus also relaxed from time to time. Had compression of the uterus been employed, unless the compression had taken place continuously for about four hours, further bleeding would have occurred, and the patient would have died very quickly.

Although immediate and continued control of hemorrhage was a *sine qua non*, prompt treatment of collapse from post-hemorrhagic shock was of equally vital importance. Plugging of the uterus, the injection of a styptic, compression of the uterus, not any of these would have remedied collapse quickly enough.

Compression of the aorta not only immediately stopped all

[1] *Practitioner*, 1907, lxxviii. 445. [2] *Difficult Labour*, 1901, p. 336.

bleeding, but also immediately influenced shock by cutting off the blood supply in the lower extremities, and so increasing the amount of blood in the heart, lungs and nerve centres. Elevation of the pelvis probably contributed greatly in this case. By this procedure, not only was venous bleeding controlled, but the blood supply to the vital centres was also maintained. The *vis a tergo* in the arterial circulation being also diminished was an important factor in helping to prevent hemorrhage after compression of the aorta ceased. Saline transfusion subcutaneously no doubt assisted materially, but without compression of the aorta would have been useless, as even with the bleeding stopped absorption was at first extremely slow, and could not have occurred at all had the bleeding not been instantly checked. Intra-venous injection of saline fluid might have succeeded, but retention of blood-plasma was of far greater value in restoring energy than would have been the introduction of mere salt solution.

Of the objections raised to compression of the aorta in the recent discussion, all had to be considered in this case with the exception of obesity. The other objections put forward were the intervention of a large uterus, inability to maintain efficient compression of the aorta,[1] damage of the sympathetic nervous system, compression of the vena cava,[2] and injury to the uterine muscle from cutting off its blood supply *via* the uterine arteries.[3]

Although this patient was not obese, she possessed unusually well-developed abdominal muscles, and in addition was highly neurotic. When the hand was placed on the abdomen to feel the uterus, previous to giving the anæsthetic, it was impossible to define the outline of the uterus properly, so great was the rigidity of the abdominal muscles. The contrast after delivery, even after the effect of the anæsthetic had disappeared, was remarkable, and was still more so when death was impending. There was, therefore, no difficulty whatever in reaching the aorta when this became necessary, although the patient when she became conscious strongly resented the proceeding. The intervention of a large uterus did not in the least prevent compression being

[1] Fitzgerald, *Practitioner*, 1906, lxxvii. 652.

[2] Duke, *Brit. M. J.*, 1907, i. 290. [3] Fitzgerald, *loc. cit.*

properly carried out, the uterus being as soft as "wet wash-leather." The fact that my assistant and myself were able to maintain efficient compression for five hours continuously is sufficient proof of the ability to maintain the necessary force long enough for effectually controlling hemorrhage. In changing compression the fist to be applied was always firmly applied on the blood-vessel below the other fist, and by this manœuvre no blood was lost in changing pressure. Injury to the sympathetic nerves, and compression of the vena cava were avoided in the one case by changing the point of pressure from time to time along the available part of the aorta, in the other by swaying the fist sideways before finally compressing the aorta. So far as could be discovered, no harm resulted in either direction.

In regard to the alleged injury to the uterus in interfering with its blood supply, quite enough blood was carried by the ovarian arteries for the nutrition of the uterine muscle, as the uterus contracted and retracted not long after compressing the aorta, and continued to do so up to the time of death. The fact that no bleeding occurred in spite of the blood-flow in the ovarian arteries continuing, proves the unimportance of considering these vessels as contributing to hemorrhage after labour.

